# Developing Nonlinear Customer Preferences Models for Product Design Using Opining Mining and Multiobjective PSO-Based ANFIS Approach

**DOI:** 10.1155/2023/6880172

**Published:** 2023-02-20

**Authors:** Huimin Jiang, Farzad Sabetzadeh, Kit Yan Chan

**Affiliations:** ^1^School of Business, Macau University of Science and Technology, Macau, China; ^2^Faculty of Business, City University of Macau, Macau, China; ^3^School of Electrical Engineering, Computing and Mathematics Sciences, Curtin University, Perth, Western Australia, Australia

## Abstract

Online customer reviews can clearly show the customer experience, and the improvement suggestions based on the experience, which are helpful to product optimization and design. However, the research on establishing a customer preference model based on online customer reviews is not ideal, and the following research problems are found in previous studies. Firstly, the product attribute is not involved in the modelling if the corresponding setting cannot be found in the product description. Secondly, the fuzziness of customers' emotions in online reviews and nonlinearity in the models were not appropriately considered. Thirdly, the adaptive neuro-fuzzy inference system (ANFIS) is an effective way to model customer preferences. However, if the number of inputs is large, the modelling process will be failed due to the complex structure and long computational time. To solve the above-given problems, this paper proposed multiobjective particle swarm optimization (PSO) based ANFIS and opinion mining, to build customer preference model by analyzing the content of online customer reviews. In the process of online review analysis, the opinion mining technology is used to conduct comprehensive analysis on customer preference and product information. According to the analysis of information, a new method for establishing customer preference model is proposed, that is, a multiobjective PSO based ANFIS. The results show that the introducing of multiobjective PSO method into ANFIS can effectively solve the defects of ANFIS itself. Taking hair dryer as a case study, it is found that the proposed approach performs better than fuzzy regression, fuzzy least-squares regression, and genetic programming based fuzzy regression in modelling customer preference.

## 1. Introduction

When researching and designing products, we need to deeply analyze the real needs of customers, such as easy to use and comfortable to hold, as they can be mapped into specific settings of the product [1]. Through the analysis of customer preference, the relationship between product specification information and customer preference can be well constructed. In the industry, the most commonly used information acquisition methods include user interviews or questionnaires, which are market analysis methods for user preferences. However, some deficiencies of them can be seen. In the stage of data collection, it is time consuming and expensive to use the method of investigation, especially when it involves interviews. On the other hand, customer surveys often only contain formatted tables or targeted interview questions. Therefore, in the survey stage, only the answers to the interview and the scores of the questionnaire can be obtained, and the data collected do not involve too much emotional expression. At present, online customer reviews with various contents can be seen in many online channels, which are very important data in the process of product planning and design or customer preference analysis and play an important role in improving the competitiveness of enterprises. Most online customer reviews are basically about their feelings in the process of using the product or their suggestions for the product. The content is written out of the customer's own will, rather than the answers to fixed preset questions. In other words, the content of these online reviews are all around products, which are difficult to obtain in many traditional survey methods. Based on the content of customer comments, it is easy to understand the customer preferences, and there is no investigation involved, which does not require the cost. However, after obtaining effective data, we need to develop and design a modelling method that can build the customer preference model.

The researchers have applied opinion mining for the content of online reviews for product optimization and design. These studies extract data from the content of online customer reviews, including product specifications, customer needs, and preferences and determine the ranking and importance according to the above dimensions. There are also some research studies on the relationship between product attributes and customer preferences, which are established by rule mining. In previous studies, three issues are noted. For the 1^st^ issue, the information on some product attributes may not be provided in the product's description and the corresponding value setting cannot be found. In this circumstance, the product attribute is usually not selected to be involved in the modelling of the relationships, even if it is related to the customer preference to a large extent. The 2^nd^ issue is about the fuzziness and nonlinearity existing in the modelling process which are not addressed well in the previous research. Online reviews have a certain relevance with the fuzziness of customers' emotional expression, but many studies have no in-depth analysis on this aspect. Also, the nonlinear relationships between customer preferences and product attributes are not expressed explicitly in the developed models. The 3^rd^ issue is found in the adaptive neuro-fuzzy inference system (ANFIS). ANFIS is mainly a multilayer feedforward network form which combines the learning power of artificial neural networks and explicit knowledge representation of fuzzy inference systems [2]. ANFIS is a widely used in modelling customer preferences to capture the fuzziness and nonlinearity in the modelling. However, if the number of inputs in ANFIS is large, the modelling process will be failed due to the complex structure and long computational time.

The research and analysis of this paper are based on the previous research content and constructively propose a new comprehensive way of multiobjective particle swarm optimization (PSO) based ANFIS and opinion mining, which can realize the modelling and analysis of customer preferences based on online customer reviews. In this paper, the opinion mining method is used to conduct a series of sentiment analyses on customer preferences and product attributes. If the value setting of product attributes cannot be found from the product description, the sentiment score of the product attribute obtained based on sentiment analysis is used as its value setting. Thus, the 1^st^ issue can be addressed. For issues 2 and 3, a multiobjective PSO-based ANFIS is proposed to model customer preferences based on the collected settings of product attributes and the information mined. ANFIS is a very effective way to establish the nonlinear relationship between customer preferences and product attributes. And, the generated fuzzy rules can address the fuzziness in the data. But when there are many inputs, ANFIS cannot model effectively. And, with the increase of input, the number of fuzzy rules grows according to the exponential speed and the complexity of the model structure greatly increases, which may lead to an increase of calculation time or training failure. To overcome the limitation of ANFIS and solve the third issue, a multiobjective PSO approach is introduced into ANFIS to determine the optimal inputs and remove the unimportant inputs to simplify the structure of ANFIS. In the multiobjective PSO approach, two objectives are involved which are minimizing modelling errors and maximizing the models' index of confidence (IC). PSO has fast convergence speed and high stability, especially in the search for the optimal solution [3]. Compared with the traditional evolutionary algorithm, the PSO algorithm can obtain a smaller error. Beiranvand et al. [4] found that multiobjective PSO algorithm has significant stability in many scenarios and has its own unique advantages in generating association rules, which is better than genetic association rules, rough PSO algorithm, multiobjective genetic algorithm, and multiobjective differential evolution algorithm. And, it is helpful to determine the best product attributes of new products.

Other contents in this paper include: Section 2 is about the related research.  Section 3 is an important chapter of this paper, which is about the proposed method in the process of customer preference modelling. In Section 4, the new modelling method in the previous chapter is applied to the actual case analysis, and then the effectiveness and practicability of the new modelling method are verified. In Section 5, the results of some validation experiments are analyzed.  Section 6 is the last section of this paper, which makes an overall summary.

## 2. Related Works

The following content is mainly about the opinion mining methods, customer preference modelling for product design in the previous studies, and the recent optimization algorithms.

### 2.1. Opinion Mining for Product Design

Opinion mining is mainly a way of computational research on the emotions and opinions expressed by text content, and it is also a way of sentiment analysis. It is a process to effectively identify the keywords and phrases related to features in the text, and then determine the emotional polarity and strength of the descriptive words. Customers' emotions can be understood through sentiment analysis in the product evaluation content published by customers, and their preferences for products can be accurately analyzed [5]. By mining the content of online customer reviews, opinion mining analysis can obtain product attributes and customer preferences and other information. The customer preference information was extracted through the establishment of an ontology learning customer demand representation system, which has better accuracy [6]. Zimmermann et al. [7] proposed a framework to discover implied product features and evaluate the content polarity of customer reviews for multiple types of products. The paper proposes a two-tier model which can use case analogy reasoning method and sentiment analysis to achieve effective identification of potential customer needs [8]. Tuarob and Tucker [9] have developed a new method to extract product features through social media automatically. In addition, a new data mining driven method is proposed, which can achieve the acquisition of customer preferences and product attribute data in a certain scale of huge social media data [10]. Zhang et al. [11] proposed an opinion mining and extraction algorithm that can discover feature opinions, opinion expressions and features. A Bayesian sampling method which can extract features from many text data is developed and designed [12]. Zhou et al. [13] developed a hybrid sentiment analysis method, which combines rough set technology and affective lexicons and realizes the enhancement of feature model by extracting online comment content information. The paper proposed a subjective and objective feature extraction in online customer reviews, mainly based on rube unsupervised rules [14]. A new case-based method was proposed, which can extract customer preferences through the integration of Kansei Engineering and text mining [15]. In order to avoid the time lag of offline surveys, Trappey et al. [16] designed a system that can determine real-time customer demand by online customer comments. Zhang et al. [17] applied the method of opinion mining in the extraction of customer preferences and product features and dealt with the redundancy of feature words by adopting a clustering technology based on semantic similarity. For product design, an ontology-based reasoning system is developed to extract effective information from online customer reviews [18]. At present, there are few papers on modelling product attributes and customer preferences based on online reviews. A rule induction framework was developed, which can form if-then rules and can associate product attributes and customer preferences of online reviews [19]. Jiang et al. [20] developed a multiobjective PSO method to obtain the association rules between product attributes and customers' emotional preferences. However, the if-then rules are not sufficient enough to be applied for the determination of the optimal product attribute design of new products.

### 2.2. Modelling Customer Preference

There is a lot of research on the methods of building customer preference models to analyze the relationship between product attributes and customer preferences. On this basis, we can better determine the optimal attribute design of new products. Much research on customer preference modelling is based on statistical techniques, such as partial least-squares analysis [21] and statistical linear regression [22]. A method based on belief rules was designed to realize the modelling process, which can determine the design elements settings of products [23]. Chen et al. [24] analyzed the relationship between product attribute design and customer preference and pointed out an artificial neural network modelling method. However, affected by the subjective judgment of the respondents, the fuzziness of the customer preferences was not considered in the above methods.

For the problem of fuzziness, researchers have done a lot of research and put forward a lot of solutions. Fung et al. [25] adopted a fuzzy inference technique to relate customer preference with the relevant product attributes. Tomasiello et al. [26] applied a variant of ANFIS with fractional Tikhonov regularization to handle problems with fuzziness. In modelling customer preference in office chair design, a method based on fuzzy rules is proposed [27]. For the analysis of the relationship between product attribute design and customer preference, a possibility regression method based on nonlinear programming is developed [28]. For building a customer preference model, a new method of Tanaka's fuzzy linear regression was developed using a survey data [29]. At present, to address the nonlinear and fuzzy problems in customer preference modelling, the methods based on polynomial modelling and fuzzy regression were proposed. A method based on genetic programming and fuzzy regression was developed for the establishment of nonlinear and fuzzy terms in structures [30]. Jiang et al. [31] introduced a chaos-based fuzzy regression approach to modelling customer preference for product design. For customer preference modelling, especially for fuzzy coefficients and polynomial structure, a fuzzy regression program based on stepwise method [32] and a fuzzy regression program based on forward selection [33] were proposed. However, the basic way in the above research is to take the traditional data survey for the modelling of customer preferences. At present, based on the information extracted from online customer reviews, there is no suitable customer preference modelling method.

### 2.3. The Recent Optimization Algorithms

The main principle of the development of PSO is based on the evolution of the social behavior of birds in biology. With the joint work of birds, they gradually converge to the specific location of food. Some variants of PSO have been proposed in recent studies. Wang et al. [34] introduced a dynamic group learning distributed particle swarm optimization for large-scale optimization and extended it for the large-scale cloud workflow scheduling. Zhang et al. [35] proposed a cooperative coevolutionary bare-bones particle swarm optimization with function-independent decomposition for a large-scale supply chain network design with uncertainties problems. Xia et al. [36] designed a triple archives PSO, which can select proper exemplars and design an efficient learning model for a particle. The social learning particle swarm optimization with a novel adaptive region search is designed to keep the diversity of the solutions and accelerate the convergence speed [37]. Li et al. [38] proposed a pipeline-based parallel particle swarm optimization which has significant potential applications in time-consumption optimization problems. For the multiobjective problem, Zhan et al. [39] proposed a novel coevolutionary technique named multiple populations for multiple objectives in which PSO is adopted for each population, and coevolutionary multiswarm PSO is developed. Liu et al. [40] proposed a coevolutionary particle swarm optimization with a bottleneck objective learning strategy to improve convergence on all objectives. Some adaptive optimization algorithms are also introduced in recent years. Zhan et al. [41] proposed an adaptive distributed differential evolution to relieve the sensitivity of strategies and parameters in complex optimization problems. Wang et al. [42] introduced an adaptive granularity learning distributed PSO with the help of machine-learning techniques to solve the problems of the slow convergence in the huge search space and the trap into local optima in large-scale optimization. Some future research directions on using evolutionary computation algorithms to solve complex continuous optimization problems are discussed in [43].

## 3. Proposed Methodology

**Figure 1:**
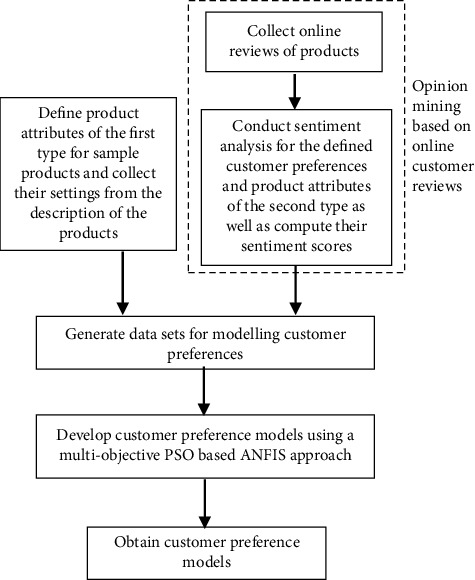
Proposed methodology.

On the basis of online customer reviews, the process of the developed method is: product feature collection, opinion mining, and customer preference model construction based on a multiobjective PSO-based ANFIS method. The process of the developed method can be seen in Figure 1. In this way, we can determine the relationship between product attributes and customer preferences and effectively solve the problem of fuzziness in the process of customer emotional expression. Product attributes used in the modelling involve two types. For the first type, the settings of the product attributes can be directly collected from the product description, while the settings of the second type cannot be obtained based on the information of the products. For the second type, the sentiment score of product attribute obtained based on sentiment analysis is used as its corresponding value setting.

### 3.1. Opinion Mining from Online Customer Review

The first is to determine the sample products. Online reviews of products can be obtained with the help of web crawler software. We collect useful data to file, such as Excel, as the data source of sentiment analysis. Then, we start to analyze the content of online customer reviews and calculate the emotional score of the second type of product attributes and customer preferences.

For the opinion mining of online reviews, this paper adopts Semantria, which has a more efficient emotional analysis function and is widely used in the industry. By importing the data collected in Excel into Semantria for analysis, we can determine the polarity of the customer's emotion and score the emotion. The specific process includes [44]: the first step: the preprocessing is used to get clean text content, from which noise in unstructured content is deleted, including stop words, punctuation and HTML characters. The second step: part of speech tagging is employed for categorizing the opinion-bearing words from online reviews into adjectives, verbs, adverbs, and nouns. Generally, nouns are used to describe product attributes and customer preferences, while adjectives and adverbs describe nouns' emotion. The third step is to analyze the content of online customer reviews and effectively extract the emotional expression content related to product attributes and customer preferences. The fourth step: feature pruning is applied to remove the incorrect features and redundant features, which involves compactness and redundancy pruning. The fifth step: the synonymous phrases are grouped into the same group, and the method of K-means clustering is adopted. The customer preference phrases of hair dryer: “excellent quality,” “great quality,” “good product,” and “high quality” were classified as group “quality.” The sixth step: sentiwordnet determines the emotional score and semantic polarity of opinion-bearing words for individual customer preferences or product attributes [45]. The final emotional score of customer preference or product attribute is then obtained based on the scores of each opinion-bearing word.

### 3.2. Modelling Customer Preference Using a Multiobjective PSO-Based ANFIS Approach

The emotional scores of the second type of product attributes and customer preferences are computed based on opinion mining. According to the settings of the first type of product attributes and the obtained emotional score, a multiobjective PSO-based ANFIS approach is developed to establish the relationship between product attributes and customer preferences. In this process, the multiobjective PSO method is used to solve the biobjective optimization problem for minimizing modelling errors and maximizing IC of the models, and the optimal input of ANFIS is obtained. The Pareto optimal solutions can be obtained, and a trade-off solution can be selected to generate a customer preference model by using ANFIS.

#### 3.2.1. Formulation of Biobjective Optimization Model

The first step is to develop the biobjective optimization model. To minimize the modelling errors, the mean absolute percentage error (MAPE) of modelling was adopted to formulate an objective function using the following equation:

(1) $$\begin{array}{c} MAPE=\frac{1}{n}\overset{n}{\underset{i=1}{\sum }}\frac{\left|{\hat{y}}_{i}-y_{i}\right|}{y_{i}}.100\text{,} \end{array}$$

where *y*_*i*_ is the *i*th sentiment score of customer preference in data sets. ${\hat{y}}_{i}$ is the *i*th predictive sentiment score based on the generated model. and *n* represents the dataset number.

*IC* is similar to the determinant confident (*R*^2^) in classical regression. The higher values of IC imply a better prediction of *y*_*i*_ [46]. The second objective function is maximizing *IC*, which is defined as follows:

(2) $$\begin{array}{c} IC=\frac{SSR}{SST}=1-\frac{SSE}{SST}\text{.} \end{array}$$

In (2), SSE = SST − SSR. SSE is the error sum of squares, SST is the total sum of squares, and SSR is the residual sum of squares, which can be calculated by (3)–(5), respectively,

(3) $$\begin{array}{c} SSE=\overset{n}{\underset{i=1}{\sum }}{\left(y_{i}-{\hat{y}}_{i}\right)}^{2}\text{,} \end{array}$$

(4) $$\begin{array}{c} SST=\overset{n}{\underset{i=1}{\sum }}{\left(y_{i}-\bar{y}\right)}^{2}\text{,} \end{array}$$

(5) $$\begin{array}{c} SSR=\overset{n}{\underset{i=1}{\sum }}{\left({\hat{y}}_{i}-\bar{y}\right)}^{2}\text{,} \end{array}$$

where $\bar{y}$ is the mean of the sentiment scores of the customer preference in the data sets.

#### 3.2.2. PSO Algorithm

In PSO, the potential solution of the problem can be regarded as a bird in the birds swarm, which is also equivalent to the “particle” in the algorithm. In the dimension of *D*, the particle will maintain its flight state at a certain speed, which is dynamically adjusted based on its flight experience and group flight experience. And, each particle will also be assigned to a fitness set, which is determined by the value of the objective function. A particle's own flight experience is its own current best position, *p*_best_, which is defined as the position with the best fitness set. At the same time, each particle also refers to the global best position, the symbol is *g*_best_, which is defined as the best value in *p*_best_. The *g*_best_ is the particle's global flying experience. The optimization search starts from the randomly initialized particle swarm and is completed by the iteration of the PSO.

In the dimension of *D*, many particles will build a particle swarm and start to search for the optimal solution continuously according to a certain speed. Each particle will adjust its position based on the global optimal position and its own optimal position, which is the basic connotation of swarm intelligence. The *i*th particle's position is set as *x*_*i*_=(*x*_*i*1_, *x*_*i*2_, ..., *x*_*id*_), at present 1 ≤ *i* ≤ *m* , 1 ≤ *d* ≤ *D* and *m* is the size of particle swarm. Also, *D* is the number of parameters to be determined for the inputs of ANFIS. The *i*th particle's speed is *v*_*i*_=(*v*_*i*1_, *v*_*i*2_, ..., *v*_*id*_). In the proposed approach, each particle represents one input set for ANFIS with a different structure to model customer preference. The specific form of particle structure can be seen in Table 1. If the value of the product attribute in a particle is 1, the product attribute is selected as the input. Otherwise, it is discarded.

At this point, if 5 is set as the number of attributes of the product and the particle value is: *x* = (0, 1, 1, 0, 1), the product attributes 2, 3, and 5 are selected as inputs, and the products attributes 1 and 4 are absent.

The *i*th particle's historical best position is *p*_*i*_=(*p*_*i*1_, *p*_*i*2_, ..., *p*_*id*_), which has the best fitness set among all the positions. The best position for the whole swarm is *p*_*g*_=(*p*_*g*1_, *p*_*g*2_, ..., *p*_*gd*_), *g* ∈ {1,2,…, *m*}. The results of *p*_*g*_ shows the optimal input set of ANFIS. According to the particle' speed, position, the distance between the global optimal position and the position at this time, and the distance between the particle's own optimal position and the position at this time, the particle's position and speed can be updated based on the idea of inertia weight [47]:

(6) $$\begin{array}{c} v_{id}^{k+1}=\omega v_{id}^{k}+c_{1}r_{1}\left(p_{id}^{k}-x_{id}^{k}\right)+c_{2}r_{2}\left(p_{gd}^{k}-x_{id}^{k}\right)\text{,}\\ x_{id}^{k+1}=x_{id}^{k}+v_{id}^{k+1}\text{,} \end{array}$$

where *x*_*id*_^*k*^ and *v*_*id*_^*k*^ represent the position vector and the speed vector of the *i*th particle at the *k*th iteration, respectively. *k* represents iterations' number. *ω* represents the inertia weight which helps the particles balance the ability of exploitation and development in the search space. *c*_1_ and *c*_2_ represent learning factors and both the values are set as 2. *r*_1_ and *r*_2_ represent random values chosen from the range [0, 1].

#### 3.2.3. Pareto Dominance

In this method, MAPE and IC are selected as the two objective functions of multiobjective PSO. According to the settings of product attributes and the results of sentiment analysis, we can obtain the data sets for customer preference modelling. Using the position vector of particles *x*_*id*_^*k*+1^ and data sets, the values of two objective functions are computed using (1) and (2) and recorded as the fitness set of each particle. A fitness set is expressed as={*f*_1_, *f*_2_}, where *f*_1_ and *f*_2_ are the values of the objective functions. With the help of the Pareto dominant theory, the multiobjective problem can be solved effectively. If a solution *x*_1_ dominates another solution *x*_2_, the following requirements should be met. From all the objective functions, we can see that the solution *x*_1_ is not worse than the solution *x*_2_. Moreover, in no less than one objective function, the solution *x*_1_ is strictly better than the solution *x*_2_. The above-given two conditions are applied to the minimization optimization problem:

(7) $$\begin{array}{c} f_{i}\left(x_{1}\right)\leq f_{i}\left(x_{2}\right),for\;all\;i\in \left\{1,2\right\}\text{,}\\ f_{j}\left(x_{1}\right)<f_{j}\left(x_{2}\right),for\;somej\in \left\{1,2\right\}\text{.} \end{array}$$

For a maximization issue, *x*_2_ is dominated by *x*_1_ if *f*_*i*_(*x*_1_) ≥ *f*_*i*_(*x*_2_), for all *f* ∈ *F* and *f*_*j*_(*x*_1_) > *f*_*j*_(*x*_2_), for at least one *f* ∈ *F*. Using Pareto dominant theory, the solution that is not dominated by other solutions can be called a Pareto optimal solution. In the process of iteration, each particle will compare its current position with its own best position. If the solution of its current position dominates its best position, the information of the best position *p*_*i*_ will be updated to be the current position. Then, based on the above-given two requirements, the solutions in *p*_*i*_ are compared with each other, and the best solution is selected as the global best position *p*_*g*_.

#### 3.2.4. ANFIS Structure

**Figure 2:**
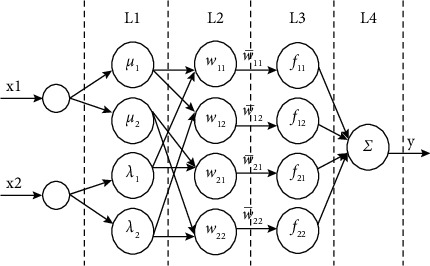
An ANFIS architecture with four layers and two inputs.

Based on the global best position *p*_*g*_, the optimal solution is obtained as the inputs of ANFIS to model customer preference. A typical ANFIS structure is shown in Figure 2. It includes two inputs and one output, and each input has two membership functions.

For each input, a membership function denotes one linguistic description. *μ*_*i*_(*x*_1_) represent the membership function for the *i*th linguistic description of *x*_1_, and *λ*_*j*_(*x*_2_) represent the membership function of the *j*th linguistic description of *x*_2_, in which *i*=1,2 and *j*=1,2. Therefore, there are four membership functions of the two inputs and they are denoted as the four nodes in the first layer (L1). The content of the triangle membership function is as follows:

(8) $$\begin{array}{c} \mu_{i}\left(x_{1}\right)=\left\{\begin{array}{cc} \frac{x_{1}-a_{i}}{b_{i}-a_{i}}\text{,} & a_{i}\leq x_{1}\leq b_{i}\text{,}\\ \frac{c_{i}-x_{1}}{c_{i}-b_{i}}\text{,} & b_{i}\leq x_{1}\leq c_{i,}\\ 0\text{,} & Otherwise\text{,} \end{array}\right.\\ \lambda_{j}\left(x_{2}\right)=\left\{\begin{array}{cc} \frac{x_{2}-s_{j}}{t_{j}-s_{j}}\text{,} & s_{j}\leq x_{2}\leq t_{j}\text{,}\\ \frac{u_{j}-x_{2}}{u_{j}-t_{j}}\text{,} & t_{j}\leq x_{2}\leq u_{j}\text{,}\\ 0\text{,} & Otherwise\text{.} \end{array}\right. \end{array}$$

In (8), (*a*_*i*_, *b*_*i*_, *c*_*i*_) and (*s*_*j*_, *t*_*j*_, *u*_*j*_) represent the triangular fuzzy numbers.

At L2, the outcome of each combination of *x*_1_ with *x*_2_ is denoted by one rule. That is, the total number of rules is 4. The fuzzy rules are described as follows:

(9) $$\begin{array}{c} R_{ij}:IF\;x_{1}\;is\;\mu_{i}\;AND\;x_{2}\;is\;\lambda_{j},THEN\;f_{ij}=p_{ij}x_{1}+q_{ij}x_{2}+r_{ij}\text{,} \end{array}$$

where *p*_*ij*_, *q*_*ij*_ with *r*_*ij*_ are the parameters of *f*_*ij*_ of fuzzy rules *R*_*ij*_. The outputs of L2 are described as follows:

(10) $$\begin{array}{c} w_{ij}=\mu_{i}\left(x_{1}\right)\lambda_{j}\left(x_{2}\right)\left(\forall i=1,2j=1,2\right)\text{.} \end{array}$$

In (10), *w*_*ij*_ is the firing strength of each fuzzy rule. The connection weight of L2 with L3 is the normalized firing strength ${\bar{w}}_{ij}$ as defined by (11). The larger the value of ${\bar{w}}_{ij}$ implies that *R*_*ij*_ is more important.

(11) $$\begin{array}{c} {\bar{w}}_{ij}=\frac{w_{ij}}{W}\;where\;W=\underset{i}{\sum }\underset{j}{\sum }w_{ij}\left(\forall i=1,2,j=1,2\right)\text{.} \end{array}$$

The internal model of *R*_*ij*_ in L3 is actually a first-order Sugeno fuzzy model, described in the following equation:

(12) $$\begin{array}{c} f_{ij}=p_{ij}x_{1}+q_{ij}x_{2}+r_{ij}\left(\forall i=1,2,j=1,2\right)\text{.} \end{array}$$

At L4, the total output is denoted by a single node and obtained by calculating the sum of all the input signals, as shown in the following equation:

(13) $$\begin{array}{c} \hat{y}=\overset{2}{\underset{i=1}{\sum }}\overset{2}{\underset{j=1}{\sum }}O_{ij}=\overset{2}{\underset{i=1}{\sum }}\overset{2}{\underset{j=1}{\sum }}{\bar{w}}_{ij}f_{ij}=\overset{2}{\underset{i=1}{\sum }}\overset{2}{\underset{j=1}{\sum }}{\bar{w}}_{ij}\left(p_{ij}x_{1}+q_{ij}x_{2}+r_{ij}\right)\text{.} \end{array}$$

It can be seen from (13) that the single output ($\hat{y}$) of ANFIS is a linear combination of all internal models under fuzzy rules and the normalized firing strengths, which is the predicted sentiment score of the customer preference.

### 3.3. Computational Procedures of the Proposed Methodology for Modelling Customer Preference Based on Online Customer Reviews

Based on online reviews, the method of building a customer preference model is as follows:

  Step 1: the product attributes of the first type are identified and their settings are collected based on the description of the products.
  Step 2: for online customer reviews of sample products, they will be collected through certain processing and stored in Excel files. In Section 3.1, there is a corresponding elaboration. Semantria will conduct a series of opinion mining for all online reviews. The definitions of the second type of product attributes and customer preferences are then completed. Based on the keywords and phrases related to each product attribute and customer preference, opinion mining is conducted again for the online reviews. The emotional scores of the product attribute and customer preferences can be obtained. According to steps 1 and 2, we can get the data sets, which can be used as the data source to simulate user preferences.
  Step 3: based on the content of Section 3.2 and the data sets in step 2, this paper proposes a multiobjective PSO-based ANFIS approach for customer preference modelling. By adopting a multiobjective PSO algorithm, ANFIS input can be determined. The group size of PSO, iteration times, the learning factor, inertia weight, and search space dimension are initialized. Based on Table 1, each particle will randomly initialize its position and speed in the corresponding range.
  Step 4: in the iteration phase, the particle's individual optimal position *p*_*i*_ and the global optimal position *p*_*g*_ of particles are initialized in the first iteration. The initial individual optimal position is set as the initial position of each particle. According to Section 3.2.4, ANFIS is used to model customer preferences, and then (15) is used to complete the overall output prediction. The values of two objective functions, MAPE and IC, are then computed for each particle using (1) and (2), respectively, and they will be the initial individual best fitness set *p*_best_. In the corresponding *p*_*i*_, particles are compared with each other using Pareto dominant theory. The particle that meets the conditions in Section 3.2.3 becomes the Pareto optimal solution and is set as the initial optimal particle. In this case, its fitness set is the initial global optimal fitness set *g*_best_ and its position vector is the initial global optimal position *p*_*g*_.
  Step 5: as the iteration process increases from *k* to *k* + 1, the particle speed vector *v*_*id*_^*k*+1^ and the position vector *x*_*id*_^*k*+1^ are updated according to (6) and (7), respectively. When the value in *x*_*id*_^*k*+1^ and *v*_*id*_^*k*+1^ is beyond the corresponding defined search range, then the value will be adjusted to the range limit value. Based on the updated position of particles, the predicted output $\hat{y}$ is obtained from ANFIS using (15). After a series of calculations for the two objective functions using (1) and (2), we can get the fitness set *F*_*i*_^*k*+1^ of ith particle after (*k*+1)th iteration. According to the Pareto dominance described in Section 3.2.3, the *p*_best_ of ith particle and *F*_*i*_^*k*+1^ can be compared. When *p*_best_ is dominated by *F*_*i*_^*k*+1^, the value of *p*_best_ is substituted by the value of *F*_*i*_^*k*+1^. At the same time, the individual optimal position of the *i*th particle will be updated to be *p*_*i*_=*x*_*id*_^*k*+1^. Among *p*_best_, the Pareto dominance is then conducted. The defined Pareto optimal solution in *p*_best_ is the new value of the global optimal fitness set *g*_best_. At the same time, the number of the optimal particle needs to be accurately recorded and its position is used as the global best position *p*_*g*_.
  Step 6: if the preset limit of iteration is met, it will stop. The *p*_*g*_ represents the optimal product attributes for modelling customer preference using ANFIS and *g*_best_ are the corresponding values of MAPE and IC. Based on equations (10), (12)∼(15), and the selected solution, the customer preference models can be established effectively and the fuzzy rules can be generated using (11).

## 4. Implementation

Based on the research of customer preference modelling, this paper adopts the method of case analysis. The research object is selected as the hair dryer, and the research work is carried out according to the hair dryers' online customer reviews. In this paper, ten products of A∼J are selected. On the Amazon shopping platform, we collected a total of 10754 published product reviews, and the review data were stored in excel through processing. Then, the process of opinion mining is implemented by Semantria. Based on the steps in Section 3.1, using the collected data, preprocessing process and part of speech tagging were conducted first. Then, phrases and keywords were extracted effectively and high frequent ones were chosen. Feature pruning was employed to delete the redundant features. After that, words and phrases which are synonymous or related to the same product attribute or customer preference were then grouped. For example, the mined phrases “easy to operate,” “separate switch,” “less noise,” “quiet,” and “simple to use” were summarized as a group “easy to use,” which is an important representative of customer preferences. Customer preferences are summarized, including five categories of quality, price, weight, easy to use, and performance. This paper analyzes the “easy to use,” which is denoted as *y* and develops a new method for customer preference modelling, that is, multiobjective PSO-based ANFIS approach. “Drying time” is one of the product attributes related to “easy to use,” which is the second type of product attribute as its settings cannot be found from the information of products and is denoted as *x*_8_. The extracted key words and phrases “quickly,” “faster,” “fast drying,” and “short time” were grouped under the category “drying time.” Among all the online reviews, the numbers of online reviews which involve customers' comments and opinions on “easy to use” and “drying time” are 304, 140, 149, 103, 88, 49, 50, 165, 81, and 64 as well as 503, 204, 250, 146, 178, 78, 70, 365, 177 and 119 for products A∼J, respectively. The online reviews were analyzed again by using the user category analysis of Semantria. Phrases and keywords associated with the product attributes of the second type and each customer's preference were treated as the settings of the “user category.” Through sentiment analysis, the sentiment scores of the product attribute of the second type and the customer preferences for each product were obtained. The examples of online reviews on “easy to use” and “drying time” as well as the obtained emotional polarity and scores are shown in Table 2. The sentiment scores of “easy to use” and “drying time” for products A∼J are obtained as shown in the last two columns in Table 3, which are used as the values of customer preference and the settings of a product attribute, respectively.

Among the product attributes of various contents, seven product attributes of the first type are related to “easy to use.” These attributes include weight attribute, length attribute, width attribute, height attribute, power attribute, heat setting attribute, and speed setting attribute, which are denoted as *x*_1_∼*x*_7_, respectively. Table 3 shows the attribute settings of 10 sample products.

With the support of the data set in Table 3, a multiobjective PSO-based ANFIS approach is proposed to construct relationships between customer preference, *y*, and product attributes *x*_1_∼*x*_8_. According to Table 1, the number of dimensions *D* in the search space of the multiobjective PSO algorithm is 8, which is equivalent to the number of product attributes. The search ranges of *x*_*id*_^*k*+1^ and *v*_*id*_^*k*+1^ of particles were [0, 1] and [−0.5, 0.5], respectively. After many operations, it can be determined that the number of iterations is 30 and the size of the particle swarm is 5, which are the minimum value settings with high prediction accuracy. From the interval of [0.1, 0.9], a random value is taken as the inertia weight *ω*. *c*_1_ and *c*_2_ are chosen as 2. *r*_1_ and *r*_2_ are randomly selected from the interval of [0, 1]. For ANFIS, the number of membership functions for each input was set as 3. Since the smallest training error was obtained at the 3^rd^ iteration and kept stable in the following iterations, the training epoch number was set as 5. The customer preference model can be established after analysis of online reviews by utilizing MATLAB software. The laptop with an i7-7500U CPU and 8 GB RAM was used as the equipment for the experiment. The optimal solutions for “easy to use” and the corresponding values of MAPE and IC were obtained and some examples are shown in Table 4. In the table, each solution represents an ANFIS structure. If the value of the product attribute is 1, the product attribute is used as the input of ANFIS. For example, the values of *x*_2_, *x*_4_, and *x*_8_ are 1 in the 7^th^ solution. Thus, the product attributes *x*_2_, *x*_4_, and *x*_8_, are used as the inputs of ANFIS for modelling customer preference.

It can be seen from the table that IC is directly proportional to the number of inputs and MAPE is inversely proportional to the number of inputs. However, based on the ANFIS structure described in Section 3.2.4, the number of terms and fuzzy rules in the model is increasing exponentially. For example, the number of fuzzy rules for one to five inputs is 3, 9, 27, 81, and 243, respectively. In other words, the calculation time and the complexity of the model increase significantly with more inputs. The modelling results in Table 4 also show when the number of inputs is equal or larger than 2, the modelling errors are very small, and the value of IC reaches the largest value of 1. To perform a trade-off between the complexity of ANFIS and modelling errors, in this study, the ANFIS with two inputs is selected to modelling customer preference because of its simple structure and good modelling accuracy. Among all the optimal solutions with two inputs, the 5^th^ optimal solution is chosen as it has the smallest MAPE and the largest *IC*. Therefore, *x*_2_ and *x*_4_ are the input attributes for ANFIS. Based on (8) and (10)∼(15), the customer preference model for “easy to use” was established based on the proposed approach.

(14) $$\begin{array}{c} 0.0107{\left(x_{2}\right)}^{2}x_{4}+0.0173x_{2}{\left(x_{4}\right)}^{2}-0.1358{\left(x_{2}\right)}^{2}-0.0894{\left(x_{4}\right)}^{2}-0.1166x_{2}x_{4}\\ \hat{y}=\frac{+0.9033x_{2}+0.0769\;x_{4}+0.0913}{1.3359x_{2}x_{4}-8.1662x_{2}-7.3539x_{4}+44.9521}\text{.} \end{array}$$

Based on the established model, the product development team can predict the customer preference score according to the new settings of product attributes. Moreover, the model can also be used to determine the optimal product attributes for designing new products by maximizing customer preference scores. Nine fuzzy rules were generated by using (9) as follows:

(15) $$\begin{array}{c} R_{11}:IF\;x_{2}\;is\;\mu_{1}\;AND\;x_{4}\;is\;\lambda_{1},THEN\;f_{11}=0.0002x_{2}+0.0001x_{4}+2.115*10^{-5}\text{,}\\ R_{12}:IF\;x_{2}\;is\;\mu_{1}\;AND\;x_{4}\;is\;\lambda_{2},THEN\;f_{12}=0.0139x_{2}+0.0225x_{4}+0.0025\text{,}\\ R_{13}:IF\;x_{2}\;is\;\mu_{1}\;AND\;x_{4}\;is\;\lambda_{3},THEN\;f_{13}=0.0022x_{2}+0.0035x_{4}+0.0004\text{,}\\ R_{21}:IF\;x_{2}\;is\;\mu_{2}\;AND\;x_{4}\;is\;\lambda_{1},THEN\;f_{21}=0.0057x_{2}+0.0044x_{4}+0.0006\text{,}\\ R_{22}:IF\;x_{2}\;is\;\mu_{2}\;AND\;x_{4}\;is\;\lambda_{2},THEN\;f_{22}=-0.22x_{2}+0.2206x_{4}-0.0041\text{,}\\ R_{23}:IF\;x_{2}\;is\;\mu_{2}\;AND\;x_{4}\;is\;\lambda_{3},THEN\;f_{23}=0.1859x_{2}-0.145x_{4}+0.0107\text{,}\\ R_{31}:IF\;x_{2}\;is\;\mu_{3}\;AND\;x_{4}\;is\;\lambda_{1},THEN\;f_{31}=0.035x_{2}+0.0276x_{4}+0.0032\text{,}\\ R_{32}:IF\;x_{2}\;is\;\mu_{3}\;AND\;x_{4}\;is\;\lambda_{2},THEN\;f_{32}=0.0038x_{2}+0.011x_{4}-0.0165\text{,}\\ R_{33}:IF\;x_{2}\;is\;\mu_{3}\;AND\;x_{4}\;is\;\lambda_{3},THEN\;f_{33}=0.0433x_{2}-0.02612x_{4}-0.0128\text{.} \end{array}$$

## 5. Validation

For the systematic analysis and evaluation of the effectiveness of the proposed method, this paper compares and analyzes the modelling results of genetic programming-based fuzzy regression (GP-FR), fuzzy least square regression (FLSR), fuzzy regression (FR), and ANFIS. In the process of building a customer preference model, it was found that ANFIS cannot be realized because of its complex structure. In order to get the value of *h* parameter in FR and FLSR, this paper selected a number of values in [0, 1] interval for experiments and tests and selected the *h* value with the lowest modelling error. The *h* value of FR was set to 0.1, and the *h* value of FLSR was set to 0.99. To make a compromise between the modelling accuracy and computational time, the models based on GP-FR were established by using a different setting of iteration number and population size. Finally, the number of iterations was set to 200 and the population size was set to 40. The generation gap, the maximum depth of the tree, and the probability of mutation and crossover were set to 0.8, 5, 0.3, and 0.7, respectively. This paper describes the settings of parameters of the proposed method in Section 4. The developed models for “easy to use” based on the four approaches and their corresponding MAPE and IC are shown in Table 5.

The table shows that all models can effectively deal with the fuzziness of modelling. However, only the models established by GP-FR and the proposed method can solve the problem of modelling nonlinearity. In addition, the value of MAPE based on the proposed method is smaller, and the value of IC is larger than those based on the other three approaches.

In order to further verify the effectiveness of the proposed method, a total of thirty validation tests are arranged. In the process of each test, two data sets were randomly selected from all data sets as testing data, and another eight data sets were used as training data for generating customer preference models. MAPE and variance of errors (VoE) in (1) and (16) are used to systematically compare and analyze the modelling results of FR, FLSR, GP-FR, and multiobjective PSO-based ANFIS.

(16) $$\begin{array}{c} VoE=\frac{1}{n-1}\overset{n}{\underset{i=1}{\sum }}{\left(\frac{\left|{\hat{y}}_{i}-y_{i}\right|}{y_{i}}.100-MAPE\right)}^{2}\text{.} \end{array}$$

**Figure 3:**
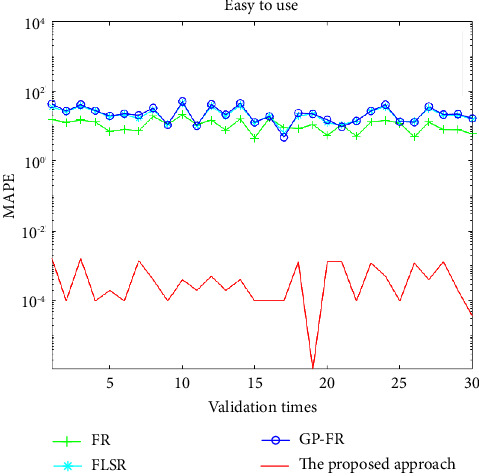
Values of MAPE based on the four approaches for validation tests.

**Figure 4:**
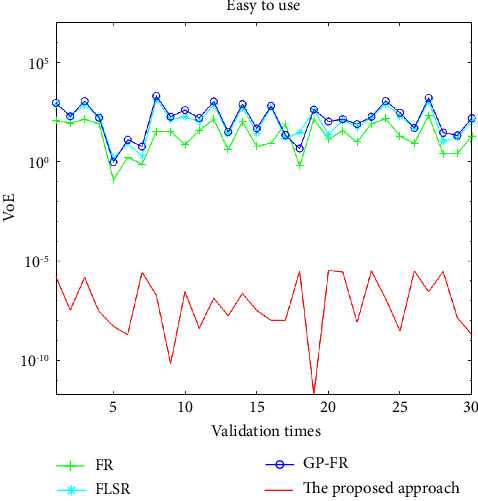
Values of VoE based on the four approaches for validation tests.

The contents in Figures 3 and 4 are about the MAPE and VoE values under the four methods, respectively. The verification results of FR, FLSR, GP-FR, and the proposed method are indicated by the line with the symbol “+,” “∗,” “O,” and the solid line, respectively.

Based on the above-given experiment settings, the training time for FR, FLSR, GP-FR, and the proposed method are 1.9186, 0.3663, 143.6772, and 1.1449 seconds, respectively. Except for the GP-FR method, it is not much different from the training time of the other three methods.

In Table 6, the average MAPE and VoE of the 30 validation tests after adopting four methods are described. Through the above-given comparative analysis, we can find that the proposed method performs better than other approaches in terms of MAPE, VoE, and their means. The mean MAPE and VoE based on the proposed approach are reduced by 10^5^ and 10^8^ times in comparison with that based on the other three approaches, respectively.

## 6. Conclusions

The traditional way to collect data is to use customer surveys. Through a series of collection and analysis of customer survey data, we can understand customers' real preferences and then establish the model. However, the process of data collection will definitely take a long time. Not only that, because the general questionnaire will set questions in advance, and the customers can answer according to the content of the questions. Therefore, the data contents collected under such characteristics do not have much emotional expression. Comparatively, online customer reviews can contain a lot of emotional expressions of customers, such as comments on products or suggestions on optimization design. In this way, customers' product preferences can be easily obtained, and there is no cost in this process. For data mining for online review content and the application in the design of new products, some research has been carried out. But in the previous research, some issues have been found as follows. Firstly, the product attributes without the setting information were not involved in the modelling of customer preference. Secondly, many research contents do not effectively solve the fuzzy problem of emotional expression in the modelling and the nonlinearity existing in the models. Thirdly, the modelling process of ANFIS will be failed if the number of inputs is large, as it leads to a complex structure and long computational time. To overcome the above research limitations, a new methodology, which involves opining mining for product attributes and customer preferences from online reviews as well as a multiobjective PSO-based ANFIS approach for establishing customer preference models, is proposed. In this paper, the corresponding application case analysis on the hair dryer products is carried out. The effectiveness and practicability of the proposed method are verified. The proposed method is compared with the ANFIS, FR, FLSR, and GP-FR approaches. By comparing the modelling results, it can be found that the model constructed by the proposed method can effectively solve the problems of nonlinearity and fuzziness. In addition, the multiobjective PSO-based ANFIS approach is superior in values of VoE and MAPE compared with other methods. In the future, we will determine the best product attributes setting of new products using the developed customer preference models. On the other hand, a study of the improvement of the proposed approach with the adaptive determination of the parameter settings for PSO and ANFIS would also be considered in future work by referring to the recent studies in Section 2.3.

## Figures and Tables

**Table 1:** Structure of a particle.

Product attribute 1	Product attribute 2	Product attribute 3	⋯	Product attribute *n*
0 or 1	0 or 1	0 or 1	0 or 1	0 or 1

**Table 2:** Examples of online reviews on “easy to use” and “drying time” with sentiment polarity and scores.

Online reviews	Sentiment polarity	Sentiment score
*Easy to use*		

… Not only was the price great, but the dryer itself is fantastic! … it gives you a fast dry and also softens your hair. The attachments it comes with easily attach to the dryer making them extremely easy to use. The cooling button does a great job setting my hair …	Positive	1.0339
Switch is all wiggly, power is relatively weak, and somewhat loud, but it has been working so far and it is inexpensive	Negative	−0.47
The dryer works very well. The only thing I don't like is the on/off switch. It is a rocker switch instead of a slider and is easy for me anyway, to turn the dryer off by mistake	Neutral	−0.0239

*Drying time*		

This hair dryer works really fast. It usually takes me 10 minutes to dry my hair in the morning and also get the straight sleek look, the hair dryer has cut that time in half	Positive	0.633
… I have thick hair and this definitely took more time than my previous dryer to dry. It did not seem to get as hot as I was accustomed to. I also had problems with my hair getting sucked in each time I used it …	Negative	−0.9869
The heat level is fine, but the highest wind or blowing speed feels like medium/light. As a male with medium-length hair, I don't see how someone could recommend this particular product to anyone with longer-than-short hair	Neutral	0.22

**Table 3:** Data sets for developing customer preference.

Product	Product attributes								Easy to use
	Weight (pounds)	Length (inches)	Width (inches)	Height (inches)	Power (wattage)	Heat setting	Speed setting	Drying time	
	*x *_1_	*x *_2_	*x *_3_	*x *_4_	*x *_5_	*x *_6_	*x *_7_	*x *_8_	
A	1.83	11	4.5	9	1875	2	2	0.2632	0.1476
B	1.5	10.8	4.3	8.5	1875	3	2	0.2416	0.0879
C	1	10.4	4.5	11	1875	3	2	0.3270	0.2132
D	2.05	10	4.2	10	1875	3	2	0.2094	0.1940
E	1	5.5	2.5	8.9	1000	0	2	0.2795	0.2475
F	1.7	11	4	9.8	1875	3	2	0.2562	0.1840
G	0.3	9.9	3.7	9.7	1875	0	3	0.2899	0.1674
H	1	8.8	3.5	11	2000	3	3	0.2745	0.0618
I	1	8.1	4.1	6.1	1875	0	3	0.1822	0.0693
J	1.8	8.5	3.5	10	2000	3	3	0.3476	0.2104

**Table 4:** Optimal solutions for “easy to use.”

Optimal solutions	*x*_1_	*x*_2_	*x*_3_	*x*_4_	*x*_5_	*x*_6_	*x*_7_	*x*_8_	MAPE	IC
1	0	0	0	1	0	0	0	0	25.0972	0.5266
2	0	0	0	0	0	0	0	1	24.0736	0.5740
3	0	0	0	1	0	0	0	1	0.0390	0.9993
4	1	0	1	0	0	0	0	0	0.0028	1
5	0	1	0	1	0	0	0	0	6.6234 *∗* 10^−4^	1
6	0	1	0	1	0	1	0	0	1.1019 *∗* 10^−5^	1
7	0	1	0	1	0	0	0	1	2.6956 *∗* 10^−6^	1
8	1	0	1	0	1	0	0	1	0.0077	1
9	0	0	1	1	0	1	1	1	2.0471 *∗* 10^−6^	1

**Table 5:** Developed models and their modelling results.

Approaches	Developed models	MAPE	IC
FR	y^=2.0411,0+−0.0977,0x1+−0.2130,0 x2+−0.5631,0x3+−0.0072,0x4+0.0032,0.0001x5+−0.2399,0x6+−1.3622,0 x7+1.74,0x8	10.4349	0.9714

FLSR	$\begin{array}{c} \hat{y}=\left(0.004,0.2842\right)+\left(0.0683,0.3281\right)x_{1}\\ +\left(-0.0115,0.0399\right)x_{2}+\left(-0.0098,0.0875\right)x_{3}\\ +\left(0.0263,0.0439\right)x_{4}+\left(0,0.0002\right)x_{5}\\ +\left(-0.0459,0.1876\right)x_{6}+\left(-0.0834,0.1286\right)x_{7}\\ +\left(0.6250,1.0635\right)x_{8} \end{array}$	21.4602	0.9330

GP-FR	$\begin{array}{c} \hat{y}=\left(-0.0556,0\right)x_{1}x_{8}+\left(0.3002,0\right)x_{4}\\ +\left(0.0360,0\right)x_{5}+\left(-0.0002,0.0002\right)x_{6}\\ +\left(-0.0343,0\right)x_{3}+\left(0.0371,0\right) \end{array}$	23.15	0.9218

Multiobjective PSO-based ANFIS	$\hat{y}=\begin{array}{c} \;0.0107\;{\left(x_{2}\right)}^{2}x_{4}+0.0173x_{2}{\left(x_{4}\right)}^{2}-0.1358\;{\left(x_{2}\right)}^{2}\\ -0.0894\;{\left(x_{4}\right)}^{2}-0.1166x_{2}x_{4}+0.9033x_{2}\\ +0.0769\;x_{4}+0.0913 \end{array}/1.3359\;x_{2}x_{4}-8.1662x_{2}-7.3539x_{4}+44.9521$	6.6234 *∗* 10^−4^	1

**Table 6:** Means of MAPE and VoE for the validation tests.

Validation errors	FR	FLSR	GP-FR	The proposed approach
MAPE	11.0212	22.4136	24.1285	5.5292 *∗* 10^−4^
VoE	49.856	279.7630	384.4177	8.433 *∗* 10^−7^

## Data Availability

The data used to support the findings of this study can be obtained from the corresponding author upon request.

## References

[1] Tseng M. M., Piller F. T. (2003). *The Customer Centric Enterprise: Advances in Mass Customization and Personalization*.

[2] Shihabudheen K. V., Pillai G. N. (2018). Recent advances in neuro-fuzzy system: a survey. *Knowledge-Based Systems*.

[3] Kennedy J., Eberhart R. Particle swarm optimization.

[4] Beiranvand V., Mobasher-Kashani M., Abu Bakar A. (2014). Multi-objective PSO algorithm for mining numerical association rules without a priori discretization. *Expert Systems with Applications*.

[5] Cambria E. (2016). Affective computing and sentiment analysis. *IEEE Intelligent Systems*.

[6] Chen X., Chen C. H., Leong K. F., Jiang X. (2013). An ontology learning system for customer needs representation in product development. *International Journal of Advanced Manufacturing Technology*.

[7] Zimmermann M., Ntoutsi E., Spiliopoulou M. (2015). Discovering and monitoring product features and the opinions on them with OPINSTREAM. *Neurocomputing*.

[8] Zhou F., Jianxin Jiao R., Linsey J. S. (2015). Latent customer needs elicitation by use case analogical reasoning from sentiment analysis of online product reviews. *Journal of Mechanical Design*.

[9] Tuarob S., Tucker C. S. (2015). Automated discovery of lead users and latent product features by mining large scale social media networks. *Journal of Mechanical Design*.

[10] Tuarob S., Tucker C. S. (2015). Quantifying product favorability and extracting notable product features using large scale social media data. *Journal of Computing and Information Science in Engineering*.

[11] Zhang H., Sekhari A., Ouzrout Y., Bouras A. (2016). Jointly identifying opinion mining elements and fuzzy measurement of opinion intensity to analyze product features. *Engineering Applications of Artificial Intelligence*.

[12] Lim S., Tucker C. S. (2016). A Bayesian sampling method for product feature extraction from large-scale textual data. *Journal of Mechanical Design*.

[13] Zhou F., Jiao J. R., Yang X. J., Lei B. (2017). Augmenting feature model through customer preference mining by hybrid sentiment analysis. *Expert Systems with Applications*.

[14] Kang Y., Zhou L. (2017). RubE: rule-based methods for extracting product features from online consumer reviews. *Information and Management*.

[15] Chiu M. C., Lin K. Z. (2018). Utilizing text mining and Kansei Engineering to support data-driven design automation at conceptual design stage. *Advanced Engineering Informatics*.

[16] Trappey A. J. C., Trappey C. V., Fan C. Y., Lee I. J. Y. (2018). Consumer driven product technology function deployment using social media and patent mining. *Advanced Engineering Informatics*.

[17] Zhang L., Chu X., Xue D. (2019). Identification of the to-be-improved product features based on online reviews for product redesign. *International Journal of Production Research*.

[18] Ali M. M., Doumbouya M. B., Louge T., Rai R., Karray M. H. (2020). Ontology-based approach to extract product’s design features from online customers’ reviews. *Computers in Industry*.

[19] Chung W., Tseng T. L. B. (2012). Discovering business intelligence from online product reviews: a rule-induction framework. *Expert Systems with Applications*.

[20] Jiang H., Kwong C. K., Park W. Y., Yu K. M. (2018). A multi-objective PSO approach of mining association rules for affective design based on online customer reviews. *Journal of Engineering Design*.

[21] Nagamachi M. (2008). Perspectives and the new trend of Kansei/affective engineering. *The TQM Journal*.

[22] You H., Ryu T., Oh K., Yun M. H., Kim K. J. (2006). Development of customer satisfaction models for automotive interior materials. *International Journal of Industrial Ergonomics*.

[23] Yang J. B., Wang Y. M., Xu D. L., Chin K. S., Chatton L. (2012). Belief rule-based methodology for mapping consumer preferences and setting product targets. *Expert Systems with Applications*.

[24] Chen C. H., Khoo L. P., Yan W. (2006). An investigation into affective design using sorting technique and Kohonen self-organising map. *Advances in Engineering Software*.

[25] Fung R. Y., Law D. S., Ip W. (1999). Design targets determination for interdependent product attributes in QFD using fuzzy inference. *Integrated Manufacturing Systems*.

[26] Tomasiello S., Pedrycz W., Loia V. (2022). On fractional Tikhonov regularization: application to the adaptive network-based fuzzy inference system for regression problems. *IEEE Transactions on Fuzzy Systems*.

[27] Park J., Han S. H. (2004). A fuzzy rule-based approach to modeling affective user satisfaction towards office chair design. *International Journal of Industrial Ergonomics*.

[28] Chen Y., Chen L. (2006). A non-linear possibilistic regression approach to model functional relationships in product planning. *International Journal of Advanced Manufacturing Technology*.

[29] Sekkeli G., Koksal G., Batmaz I., Türker Bayrak Ö. (2010). Classification models based on Tanaka’s fuzzy linear regression approach: the case of customer satisfaction modeling. *Journal of Intelligent and Fuzzy Systems*.

[30] Chan K. Y., Kwong C. K., Dillon T. S., Fung K. Y. (2011). An intelligent fuzzy regression approach for affective product design that captures nonlinearity and fuzziness. *Journal of Engineering Design*.

[31] Jiang H., Kwong C. K., Ip W. H., Chen Z. (2013). Chaos-based fuzzy regression approach to modeling customer satisfaction for product design. *IEEE Transactions on Fuzzy Systems*.

[32] Chan K. Y., Lam H. K., Dillon T. S., Ling S. H. (2015). A stepwise-based fuzzy regression procedure for developing customer preference models in new product development. *IEEE Transactions on Fuzzy Systems*.

[33] Chan K. Y., Ling S. H. (2016). A forward selection based fuzzy regression for new product development that correlates engineering characteristics with consumer preferences. *Journal of Intelligent and Fuzzy Systems*.

[34] Wang Z. J., Zhan Z. H., Yu W. J. (2020). Dynamic group learning distributed particle swarm optimization for large-scale optimization and its application in cloud workflow scheduling. *IEEE Transactions on Cybernetics*.

[35] Zhang X., Du K. J., Zhan Z. H., Kwong S., Gu T. L., Zhang J. (2020). Cooperative coevolutionary bare-bones particle swarm optimization with function independent decomposition for large-scale supply chain network design with uncertainties. *IEEE Transactions on Cybernetics*.

[36] Xia X., Gui L., Yu F. (2020). Triple archives particle swarm optimization. *IEEE Transactions on Cybernetics*.

[37] Jian J. R., Chen Z. G., Zhan Z. H., Zhang J. (2021). Region encoding helps evolutionary computation evolve faster: a new solution encoding scheme in particle swarm for large-scale optimization. *IEEE Transactions on Evolutionary Computation*.

[38] Li J. Y., Zhan Z. H., Liu R. D., Wang C., Kwong S., Zhang J. (2021). Generation-level parallelism for evolutionary computation: a pipeline-based parallel particle swarm optimization. *IEEE Transactions on Cybernetics*.

[39] Zhan Z. H., Li J., Cao J., Zhang J., Chung H. S. H., Shi Y. H. (2013). Multiple populations for multiple objectives: a coevolutionary technique for solving multiobjective optimization problems. *IEEE Transactions on Cybernetics*.

[40] Liu X. F., Zhan Z. H., Gao Y., Zhang J., Kwong S., Zhang J. (2019). Coevolutionary particle swarm optimization with bottleneck objective learning strategy for many-objective optimization. *IEEE Transactions on Evolutionary Computation*.

[41] Zhan Z. H., Wang Z. J., Jin H., Zhang J. (2020). Adaptive distributed differential evolution. *IEEE Transactions on Cybernetics*.

[42] Wang Z. J., Zhan Z. H., Kwong S., Jin H., Zhang J. (2021). Adaptive granularity learning distributed particle swarm optimization for large-scale optimization. *IEEE Transactions on Cybernetics*.

[43] Zhan Z. H., Shi L., Tan K. C., Zhang J. (2022). A survey on evolutionary computation for complex continuous optimization. *Artificial Intelligence Review*.

[44] Lexalytics (2021). Lexalytics. https://www.lexalytics.com.

[45] Baccianella S., Esuli A., Sebastiani F. SentiWordNet 3.0: an enhanced lexical resource for sentiment analysis and opinion mining.

[46] Azadeh A., Seraj O., Saberi M. (2011). An integrated fuzzy regression–analysis of variance algorithm for improvement of electricity consumption estimation in uncertain environments. *International Journal of Advanced Manufacturing Technology*.

[47] Shi Y., Eberhart R. A modified particle swarm optimizer.

